# *De novo* transcriptome sequencing of radish (*Raphanus sativus* L.) and analysis of major genes involved in glucosinolate metabolism

**DOI:** 10.1186/1471-2164-14-836

**Published:** 2013-11-27

**Authors:** Yan Wang, Yan Pan, Zhe Liu, Xianwen Zhu, Lulu Zhai, Liang Xu, Rugang Yu, Yiqin Gong, Liwang Liu

**Affiliations:** National Key Laboratory of Crop Genetics and Germplasm Enhancement, College of Horticulture, Nanjing Agricultural University, Nanjing, 210095 P.R. China; Engineering Research Center of Horticultural Crop Germplasm Enhancement and Utilization, Ministry of Education of P.R. China, College of Horticulture, Nanjing Agricultural University, Nanjing, 210095 P.R. China; Institute of Vegetable Crops, Wenzhou Academy of Agricultural Sciences, Wenzhou Vocation College of Science & Technology, Wenzhou, 325014 P.R. China; Department of Plant Sciences, North Dakota State University, Fargo, ND 58108 USA

**Keywords:** Radish, *De novo* assembly, RNA-Seq, Transcriptome, Glucosinolate metabolic pathways

## Abstract

**Background:**

Radish (*Raphanus sativus* L.), is an important root vegetable crop worldwide. Glucosinolates in the fleshy taproot significantly affect the flavor and nutritional quality of radish. However, little is known about the molecular mechanisms underlying glucosinolate metabolism in radish taproots. The limited availability of radish genomic information has greatly hindered functional genomic analysis and molecular breeding in radish.

**Results:**

In this study, a high-throughput, large-scale RNA sequencing technology was employed to characterize the *de novo* transcriptome of radish roots at different stages of development. Approximately 66.11 million paired-end reads representing 73,084 unigenes with a N50 length of 1,095 bp, and a total length of 55.73 Mb were obtained. Comparison with the publicly available protein database indicates that a total of 67,305 (about 92.09% of the assembled unigenes) unigenes exhibit similarity (e –value ≤ 1.0e^-5^) to known proteins. The functional annotation and classification including Gene Ontology (GO), Clusters of Orthologous Group (COG) and Kyoto Encyclopedia of Genes and Genomes (KEGG) analysis revealed that the main activated genes in radish taproots are predominately involved in basic physiological and metabolic processes, biosynthesis of secondary metabolite pathways, signal transduction mechanisms and other cellular components and molecular function related terms. The majority of the genes encoding enzymes involved in glucosinolate (GS) metabolism and regulation pathways were identified in the unigene dataset by targeted searches of their annotations. A number of candidate radish genes in the glucosinolate metabolism related pathways were also discovered, from which, eight genes were validated by T-A cloning and sequencing while four were validated by quantitative RT-PCR expression profiling.

**Conclusions:**

The ensuing transcriptome dataset provides a comprehensive sequence resource for molecular genetics research in radish. It will serve as an important public information platform to further understanding of the molecular mechanisms involved in biosynthesis and metabolism of the related nutritional and flavor components during taproot formation in radish.

**Electronic supplementary material:**

The online version of this article (doi:10.1186/1471-2164-14-836) contains supplementary material, which is available to authorized users.

## Background

Radish (*Raphanus sativus* L.) is an annual or biennial herb of the *Brassicaceae* family, and it is an economically important root vegetable crop produced throughout the world [1, 2]. The edible part of radish is its taproot, which is an excellent source of carbohydrates, dietary fiber, and essential mineral and organic nutrients to human beings [3–5]. Radish roots also contain valuable phytochemicals and have been used for many medicinal purposes [6, 7]. For example, the roots are a rich source of glucosinolates (GS) [8]. GS and their breakdown products such as isothiocyanates (ITC) are secondary metabolites widely present in the *Brassicaceae* family. The ITC contribute to the flavor and taste of the *Brassicaceae* vegetables as an important ingredient and have anti-carcinogenic properties [8, 9].

The formation and development of taproot is a complex morphogenetic process controlled by interactions among genetic, environmental and physiological factors [1, 10–12]. Essentially, fleshy root formation is a result of selective expression of related genes. However, the lack of genomic information impedes our understanding of the molecular mechanisms underlying taproot development. Recent analysis of transcript differences between two cDNA libraries from the early and late seedling developmental stages have demonstrated that a set of genes involved in starch and sucrose metabolism, and in phenylpropanoid biosynthesis may be the dominant metabolic pathways during the early stages of taproot formation in radish [13]. This has enabled the mining of genes that are possibly involved in taproot development. However, the molecular mechanisms involved in biosynthesis and metabolism of the related nutritional and flavor components during taproot formation are not well known, especially for many secondary metabolites such as glucosinolates.

Next-generation sequencing (NGS) -based RNA sequencing for transcriptome methods (RNA-seq) allows simultaneous acquisition of sequences for gene discovery as well as transcript identification involved in specific biological processes. This is especially suitable for non-model organisms whose genomic sequences are unknown [14–16]. In recent years, RNA-seq has emerged as a powerful method for discovering and identifying genes involved in biosynthesis of various secondary metabolites, such as, carotenoid biosynthesis in *Momordica cochinchinensis*[17], cellulose and lignin biosynthesis in Chinese fir [18], tea-specific compounds i.e. flavonoid, theanine and caffeine biosynthesis pathways in tea [19], biosynthesis of flavonoid in Safflower [20], biosynthesis of active ingredients in *Salvia miltiorrhiz*a [21] and biosynthesis of capsaicinoid in chili pepper [22].

Glucosinolate content is a main trait of radish cultivars and is important for flavor formation and nutritional quality of the taproot [8, 9]. Previous studies mainly focused on developing analysis methods to determine GS content in radish, and also to determine variation in GS composition or content in different cultivars, growing conditions, and growth stages [8, 23, 24]. Furthermore, three candidate genes for controlling the GS content in radish roots were identified from single nucleotide polymorphism (SNP) markers developed with GS [25]. However, molecular mechanisms underlying GS metabolism in radish still require elucidation, especially for identification of the full set of genes involved in these related pathways.

In the present study, NGS-based Illumina paired-end solexa sequencing platform was employed to characterize the fleshy taproot *de novo* transcriptome in radish. A large set of radish transcript sequences were obtained to discover the majority of the activated genes involved in radish taproot. The candidate genes involved in the glucosinolate metabolism and regulation were successfully identified in radish. The sequence of representative genes and expression patterns were further validated. The root *de novo* transcriptome was comprehensively characterized in radish. This would provide a public information platform for understanding the molecular mechanisms involved in the metabolism of nutritional and flavor components during taproot formation, and facilitate the genetic improvement of quality traits in radish molecular breeding programs.

## Results and discussion

### Illumina sequencing and *de novo* assembly of radish root transcriptome

To develop a comprehensive overview of the radish root transcriptome, a cDNA library denoted as ‘CKA’, prepared from three mixed RNA samples from taproots at different stages of development (seedling, taproot thickening, and mature stages) was subjected to pair-end read (PE) sequencing with the Illumina platform. It has been reported that PE sequencing not only increases the depth of sequencing, but also improve *de novo* assembly efficiency [18, 26]. After removing the reads with adaptors, reads with unknown nucleotides larger than 5% and low quality reads, 66,110,340 clean PE reads consisting of 5,949,930,600 nucleotides (nt) were obtained with an average GC content of 47.34% (Table 1). The output was similar to a previous study on radish transcriptome from two root cDNA libraries, which generated a total of 53.6 million and 53.7 million clean reads, respectively [13]. All high-quality clean reads were assembled into 150,455 contigs with an average length of 299 bp, and the length distribution of the assembled contigs was as shown in Additional file 1A. The contigs were further joined into 73,084 unigenes with a N50 length of 1095 bp, and a total length of 55.73 Mb using paired-end information and gap-filling process (Table 2). Majority of the unigenes ranged from 300 to 1500 bp, and accounted for 88.30% of all unigenes (64,418) (Additional file 1B).

**Table 1:** **Statistics of output sequencing**

Samples	CKA
Total raw reads	71,947,118
Total clean reads	66,110,340
Total clean nucleotides (nt)	5,949,930,600
Q20 percentage	97.79%
N percentage	0.00%
GC percentage	47.34%

**Table 2:** **Statistics of assembly quality**

	Contig	Unigene
Total number	150,455	73,084
Total length (nt)	44,968,854	55,733,722
Mean length (nt)	299	763
N50	458	1095
Total consensus sequences	-	73,084
Distinct clusters	-	38,040
Distinct singletons	-	35,044

### Functional annotation and classification of the assembled unigenes

**Figure 1:**
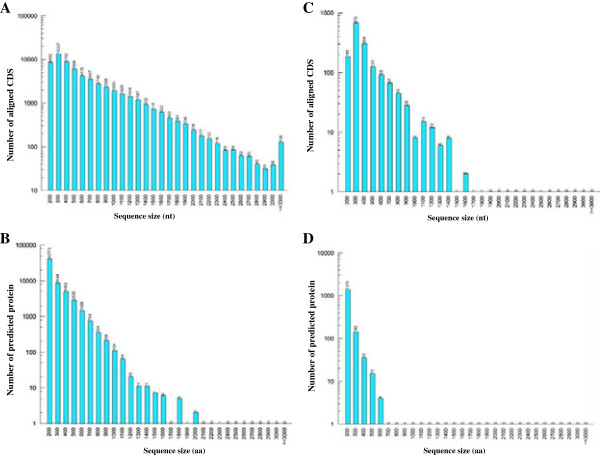
**The length distribution of the coding sequence (CDS) and predicted proteins by BLASTX and ESTScan software from the unigenes. A.** Aligned CDS by BLASTX. **B.** Predicted proteins by BLASTX. **C.** Aligned CDS by ESTScan. **D.** Predicted proteins by ESTScan.

In total, 67,305 (92.09% of all unigenes) unigenes significantly matched a sequence in at least one of the public databases including NCBI non-redundant protein (Nr), Gene Ontology (GO), Clusters of Orthologous Groups (COGs), Swiss-Prot protein and the Kyoto Encyclopedia of Genes and Genomes (KEGG) (Table 3). The rate of annotated unigenes was higher than the range of previously studies in other non-model species (73.6% in blueberry, 58% in safflower flowers and 58.01% in Chinese fir), indicating their integrity and the relatively conserved functions of the assembled transcript sequences in radish [18, 20, 27]. The size distribution of the BLAST-aligned coding sequence (CDS) and predicted proteins are shown in Figure 1A, B, respectively. The remaining 7.91% of unigenes (5,779) that did not match sequences in the databases were analyzed by ESTScan to predict coding regions. An additional 1,573 unigenes (2.15%) also showed orientation in the transcriptome coding sequence (Figure 1C, D). The sequences without a homologous hit may represent novel genes specifically expressed in radish root; or they could be attributed to other technical or biological biases, such as assembly parameters. Furthermore, some cDNAs are non-coding, lineage-specific or highly variable, which need to be further verified [27–29].

**Table 3:** **Summary statistics of functional annotation for radish root unigenes in public databases**

Public protein database	No. of unigene hit	Percentage (%)
NR	61,513	84.17
SwissProt	38,946	53.29
KEGG	33,567	45.93
COG	19,888	27.21
GO	52,572	71.93
ALL	67,305	92.09

**Figure 2:**
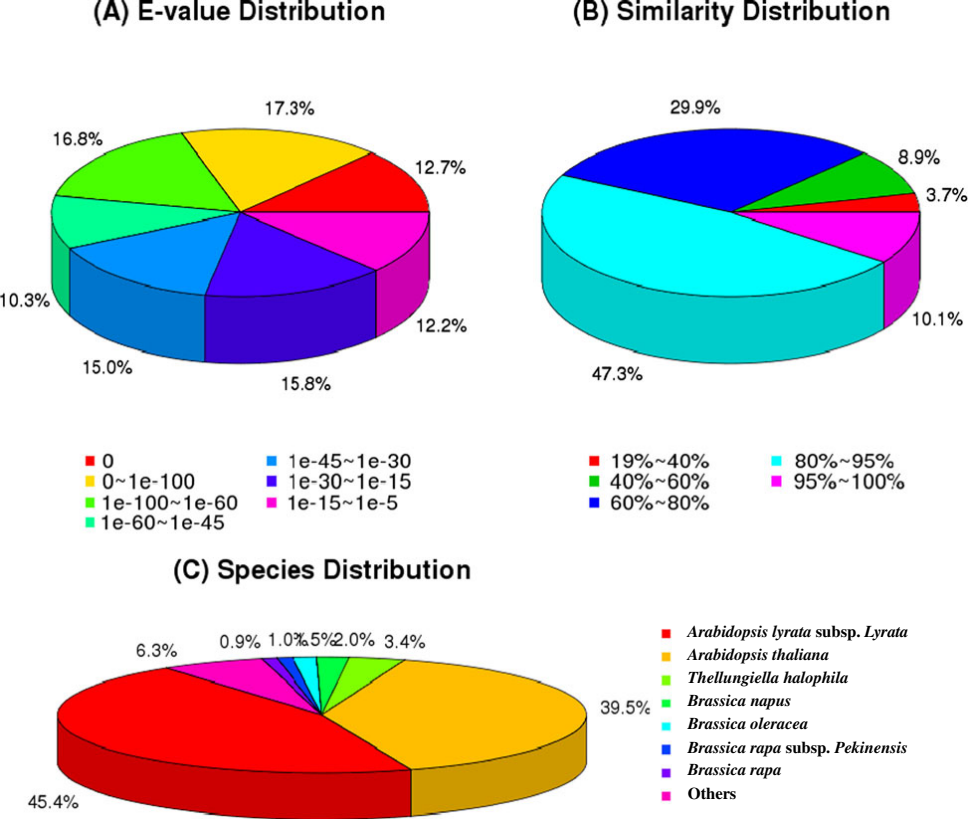
**Characteristics of sequence homology of radish root BLASTED against NCBI non-redundant (NR) database. A.** E-value distribution of BLAST hits for matched unigene sequences, using an E-value cutoff of 1.0E^-5^. **B.** Similarity distribution of top BLAST hits for each unigene. **C.** Species distribution of the top BLAST hits.

For the nr annotations, 61,513 of the unigenes (84.17%) were found to be matched in the database. Further analysis of the BLAST data indicated that 57.06% of the top hits showed strong homology with the E-value < 1.0e^-45^, while 65.47% of the matched sequences showed moderate homology with the E-value between 1.0e^-5^and 1.0 e^-45^ (Figure 2A). The identity distribution pattern showed that 57.42% of the sequences had a similarity higher than 80%, while 42.28% showed similarity between 19% and 80% (Figure 2B). The majority of the annotated sequences corresponded to the known nucleotide sequences of plant species, with 45.44%, 39.47%, 3.41%, 1.98% and 1.45% matching with *A. lyrata subsp. Lyrata*, *A. thaliana*, *Thellungiella halophila*, *B. napus* and *B. oleracea*, respectively (Figure 2C). All the top five species with BLAST hits belonged to the *Brassicaceae* family, implying that the sequences of the radish transcripts obtained in the present study were assembled and annotated properly [30].

**Figure 3:**
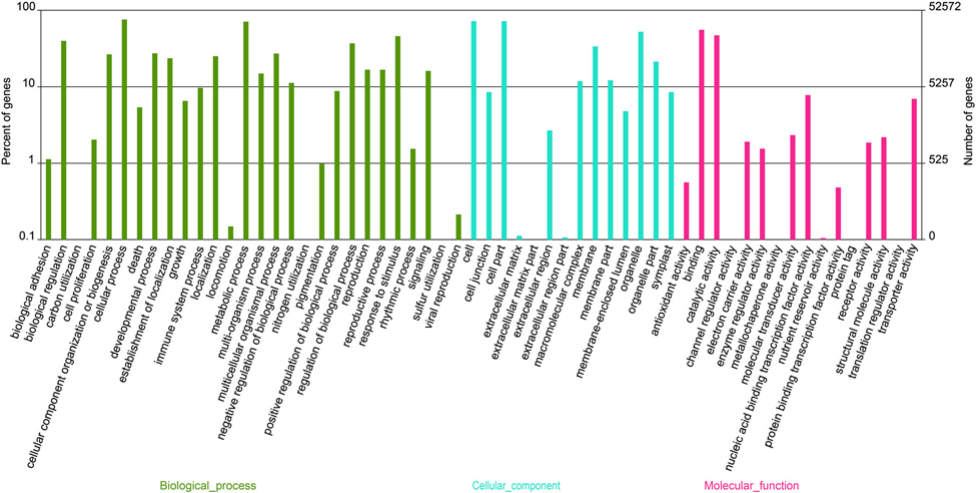
**Gene ontology classification of the CKA- unigene.**

GO annotation is an international classification system that can provide standardized vocabulary for assigning functions of the uncharacterized sequences [31]. BLAST2GO program was used to get GO terms for all assembled unigenes and a total of 52,572 unigenes (71.93% of all the assembled unigenes) were assigned at least one GO term. In many cases, multiple terms were assigned to the same transcript, and all the GO terms were classified into 58 functional groups including biological processes, cellular component, and molecular function at the second level (Figure 3). Among biological processes, transcript sequences assigned to cellular (39,716) and metabolic processes (37,191) were the most abundant. Within the molecular function category, the majority of the GO terms were predominantly assigned to binding (29,099) and catalytic activity (24,635). For cellular components, those assignments were mostly given to cell (37,809) and cell part (37,803). The findings revealed that the main GO classifications involved in the annotated unigenes were responsible for fundamental biological regulation and metabolism. These results were concurrent with a previously reported study of *de novo* transcriptome analysis in tuberous root of sweet potato [28].

**Figure 4:**
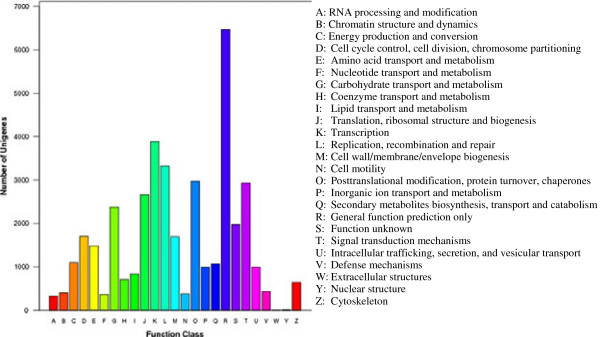
**COG function classification of the CKA- unigene.**

Every protein in the COG database is assumed to be evolved from an ancestor, and the whole database is built on coding proteins with complete genomes as well as system evolution relationships of bacteria, algae and eukaryotes [32, 33]. Overall, 19,888 of 73,084 (27.21%) unigenes were assigned to the COG classification (Table 3). Since some of these unigenes were annotated with multiple COG functions, a total of 39,787 functional annotations were produced. Among the 25 COG categories, the cluster for ‘general functions prediction only’ (6,468, 16.26%) associated with basic physiological and metabolic functions represented the largest group, followed by ‘Transcription’ (3,889, 9.77%), ‘Replication, recombination and repair’ (3,326, 8.36%), ‘Post-translational modification, protein turnover, chaperones’ (2,974, 7.47%), and ‘Signal transduction mechanisms’ (2,937, 7.38%), whereas only few unigenes were assigned to ‘Extra cellular structures’ and ‘Nuclear structure’ (Figure 4).

**Figure 5:**
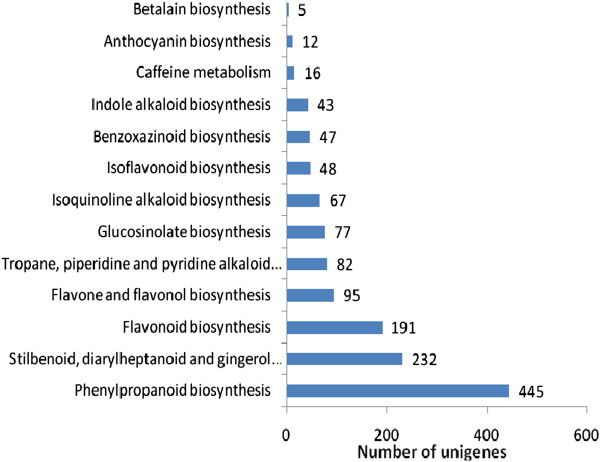
**Classification based on categories of secondary metabolite biosynthesis.**

KEGG pathway database can facilitate to systematically understand the biological functions of genes in terms of networks [21, 32]. To identify the biological pathways activated in radish roots, the assembled unigenes were annotated with KEGG Orthology (KO) numbers using BLASTx alignments against KEGG with a cut-off E value of 10^-5^. A total of 33,567 unigenes were significantly matched in the database, and were assigned to 128 KEGG pathways. The result showed that the five largest pathway groups were metabolic pathways [ko01100, 7,391(22.02%)], biosynthesis of secondary metabolites [ko01110, 3,363 (10.02%)], plant hormone signal transduction [ko04075, 1,928(5.74%)], plant-pathogen interactions [ko04626, 1,925(5.74%)] and RNA transport [ko30313, 1,285(3.83%)] (Additional file 2). In metabolism categories, the biosynthesis of secondary metabolites represented the most predominant pathways, which were sorted into 13 subcategories including phenylpropanoid biosynthesis, glucosinolate biosynthesis, flavonoid biosynthesis, betalain biosynthesis and some others (Figure 5). These annotations of gene or protein names and descriptions, gene ontology terms, putative conserved domains, and potential metabolic pathways would provide a valuable resource for investigating specific processes, functions and pathways involved in radish taproot development. These genes involved in the enrichment of secondary metabolite biosynthesis related pathways would greatly enhance the potential utilization of the radish root in nutrition and pharmacy.

### Identification of candidate genes involved in the glucosinolate metabolism of radish

**Figure 6:**
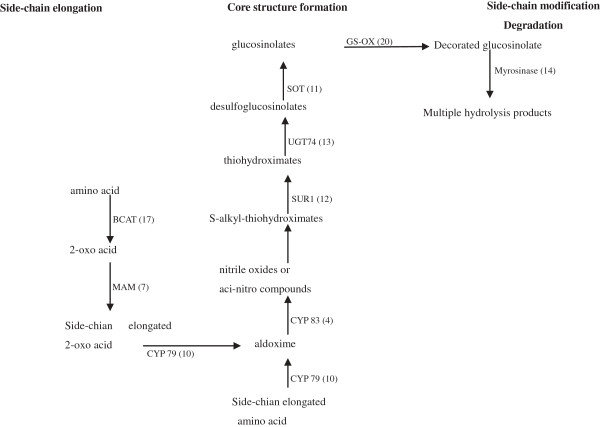
**Assembled radish unigenes that may be involved in the glucosinolates metabolism pathway.** The numbers in brackets following each gene name indicate the number of transcritome unigenes annotated to that gene.

In the past decade, the main pathway of glucosinolate (GS) biosynthesis has been well understood in *A. thaliana* and *B. rapa*, and many critical genes have been successfully discovered and functionally characterized [34, 35]. The biosynthesis of GS is generally divided into three independent phases: (i) amino acid side-chain elongation of selected precursor amino acids (only Met and Phe), (ii) core structure formation, (iii) and subsequent side-chain modification [36–38]. According to the currently accepted GS biosynthetic pathways in *A. thaliana* and *B. rapa*, a total of 94 unigenes in our transcriptome dataset were found to be homologous to the previously identified genes encoding all of the eight related enzymes of all three phases. The result indicated that this pathway was rather well conserved in *Brassicaceae* family. Furthermore, 14 unigenes were found to be homologous to the genes encoding myrosinase, which is a critical functional enzyme involved in the GS degradation (Figure 6 and Additional file 3). In most cases, more than one unique sequence was annotated as encoding the same enzyme. Such sequences may represent different fragments of a single transcript, different members of a gene family, or both [17, 39].

Initially, the parent amino acid is deaminated to form the corresponding 2-oxo acid by a branched-chain amino acid aminotransferase (BCAT, K00826, EC: 2.6.1.42). In *A. thaliana*, there are seven genes encoding the BCATs, and it is known to be fairly well conserved [40]. In our annotated radish transcriptome unigene dataset, 17 sequences corresponding to five homologous BCAT genes (BCAT 2–5) were successfully identified. Subsequently, methylthioalkylmalate synthase (MAM, K15741, EC: 2.3.3.-) catalyzes 2-oxo acid condense with acetyl-CoA to yield a 2-oxo acid with one more methylene group (−CH2–) than the starting compound. Hereupon, the elongated 2-oxo acid can enter the core glucosinolate structure pathway or proceed through another round of chain elongation. Seven sequences encoding MAM were discovered in our transcriptomic analysis.

The formation of primary glucosinolates involved in core structure biosynthesis is accomplished through five different biochemical steps that synthesize several intermediates. It begins with the oxidation of the precursor amino acids to aldoximes by cytochromes P450 belonging to the CYP79 family, which is composed of a number of catalytic subfamilies. Genome analyses have revealed that *Arabidopsis* contains seven different CYP79 genes (i.e. CYP79A1, B1, B2, B3, F1, F2 and F3) [41]. In the current study, ten unigene sequences were identified corresponding to the seven different genes with a high homology to CYP79s. All these seven gene members in the *Arabidopsis* genome were also identified in the radish transcriptome, which further confirmed the close relationship between these two species. Aldoximes are further oxidized to activated compounds (either nitrile oxides or aci-nitro compounds) by cytochromes P450 of the CYP83 family. Based on sequence similarities, four unigenes were identified corresponding to the two CYP83 genes (*CYP83A1* and *CYP83B1*). The activated aldoximes are conjugated with cysteine as a sulfur donor to produce S-alkyl-thiohydroximates; however, it is not clear whether this conjugation is enzyme-mediated. The S-alkylthiohydroximate conjugates are converted to thiohydroximates by the C-S lyase SUPERROOT1 (SUR1, K11819) [42]. In the present study, 12 homolog sequences were discovered encoding SUR1. Thiohydroximates are in turn S-glucosylated by glucosyltransferases of the UGT74 family to form desulfoglucosinolates. Overall, 13 unigenes were identified as UGT74s including UGT74B1, C1, F1 and F2. The final step in the synthesis of the GS core structure was catalyzed by desulfoglucosinolate sulfotransferase (SOT, K11821). There are three close homologous SOT genes (SOT16, 17and18), which were identified in *Arabidopsis* to catalyze this reaction with a wide variety of desulfoglucosinolate substrates [43]. A total of 11 unigenes from our RNA-seq dataset were identified as SOTs including all three homologies found in *Arabidopsis.*

The initially produced parent glucosinolate from core structure is subject to a wide range of side chain modifications, which entail various kinds of reactions including oxidations, eliminations, alkylations, and esterifications. Kliebenstein et al. (2011) identified three genes responsible for side chain modification of aliphatic glucosinolates in *Arabidopsis* by QTL analyses [44], named GS-OX, GS-AOP and GS-OH; and functionally characterized two genes including *AOP2, AOP3* of the GS-AOP cluster. In this study, 20 unigenes ranging from 252 bp to 1,921 bp were homologous to the genes encoding GS-OX; however, the other genes corresponding to the modification of side chain could not be identified.

Upon plant damage, the GS can be degraded to a variety of hydrolysis products such as isothiocyanates, oxazolidine-2-thiones, nitriles, epithionitriles, and thiocyanates. The hydrolytic process is catalyzed by a Beta-thioglucoside glucohydrolase (myrosinase, EC 3.2.3.1, K01188). Until now, myrosinase genes have been isolated from many plant species such as turnip, *A. thaliana* and mustard , which indicated that these genes are encoded by a multigene family and were classified into four subtypes(MA, MB, MC and TGG) on the basis of amino acid sequences [45]. Additionally, two cDNA clones of myrosinase were isolated from radish seedlings, and both of them were identified as B type myrosinases [46]. In this study, 14 unigenes were found which were homologs of genes encoding myrosinase, and most of them were predicted as MB subtypes.

### Identification of genes involved in MYB transcription factors

MYB transcription factors represent a family of proteins that include the conserved MYB DNA-binding domain, which can control diverse pathways and processes corresponding to plant secondary metabolism [47, 48]. It was reported that many members of the MYB family could regulate the expression of related genes at the transcriptional level to control the process of GS metabolism in *A. thaliana*. For example, MYB28, 29 and 76 exerted a specific and coordinated control on the regulation of aliphatic GS biosynthesis, while MYB34, 51 and 122 could regulate the synthesis of indolic GS [49, 50]. From our radish transcriptome analysis, a total of 257 unigenes were predicted to code MYB proteins including a large number of members (i.e., MYB 2, 3, 4, 25, 28, 29, 43, 47, 52, 56, 58, 65, 69, 73, 78, 95, 103, 108, 121, etc.) (Additional file 4). However, the specific function of the particular MYB member in GS metabolism of radish need to be further verified with functional genomics approach.

### Validation and expression analysis of genes involved in GS metabolism

To check the quality of the assembly and annotation data from the Solexa sequencing, full-length cDNA sequences of eight selected genes from glucosinolate metabolism and regulation process were isolated by T-A cloning with the Sanger method and compared with the assembled sequences. The length of these genes varied from 1,086 bp to 1,641 bp (Table 4). Overall, the assembled unigenes covered more than 95% of the corresponding full-length genes and two of them were predicted to contain the complete ORF. Additionally, the sequence variation was minimal (> 98% pairwise identity), which validated the NGS-based RNA-seq procedures was reliable.

**Table 4:** **Sequence analyses of the eight putative radish genes involved in glucosinolate metabolism process**

Gene	Full-length cDNA	Number	Coverage	ORF similarity	Gap
*RsBCAT4*	1086	1	96.70%	99.53%	3.25%
*RsCYP79F1*	1623	2	98.58%	99.94%	0.73%
*RsCYP83A1*	1506	2	99.60%	99.80%	0.39%
*RsSUR1*	1371	3	100%	99.93%	5.30%
*RsUGT74B1*	1386	2	95.56%	98.89%	4.44%
*RsGS-OX1*	1380	2	100%	99.80%	0.39%
*RsMYB28*	1092	1	99.45%	98.74%	0.54%
*RsMyr1*	1647	1	99.81%	99.52%	0.24%

**Figure 7:**
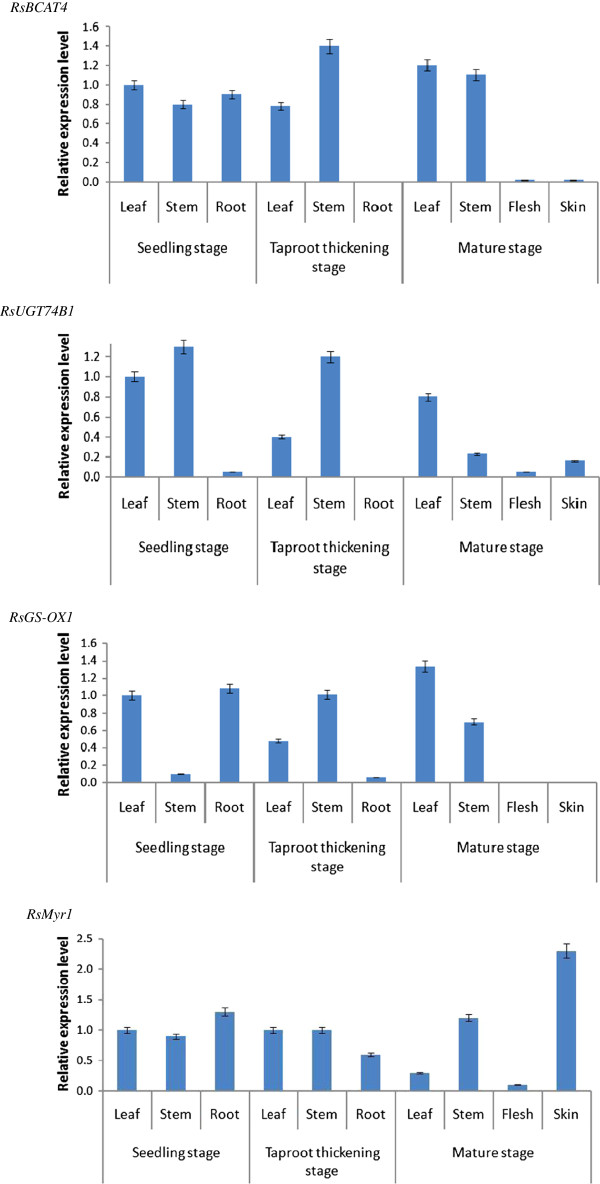
**qRT-PCR expression analysis of four selected gene expression levels in different tissues during three developmental stages in radish.**

The qRT-PCR analysis was used to compare the dynamic expression patterns of four selected genes, *RsBCAT4, RsUGT74B1, RsGS-OX1* and *RsMyr1*, in different organs at three developmental stages. It was reported that several genes involved in the GS metabolism showed distinct spatiotemporal expression patterns in different species such as *BCAT* gene in *A. thaliana*[51]*, and Myr* gene *in B. napus*[52], horseradish [53], and radish [46]. As shown in Figure 7, the expression of all these four genes in radish roots exhibited variations among different organs from different stages. *RsBCAT4* was expressed weakly in root (including flesh and skin) at taproot thickening and mature stage, and the remaining samples showed inconspicuous changes. *RsUGT74B1* exhibited higher expression in leaf and stem at seedling stage, and in stem at taproot thickening stage, whereas weaker expression was observed in root at all developmental stages. The expression of *RsGS-OX1* in root decreased in the following order: seedling, taproot thickening, and mature stage. Obvious changes in the expression level of *RsMyr1* were observed among organs at mature stages (fresh > stem > leaf > skin), but exhibited inconspicuous variations at the other two stages.

## Conclusions

In this study, NGS-based Illumina paired-end solexa sequencing platform was employed to characterize the fleshy taproot *de novo* transcriptome in radish. Approximately 66.11 million paired-end reads representing 73,084 unigenes with a N50 length of 1,095 bp, and a total length of 55.73 Mb were obtained. A total of 67,305 unigenes were successfully annotated by blastx analysis using the publicly available protein database. It was revealed that the main genes activated in radish taproot, were predominately involved in basic physiological and metabolic processes, biosynthesis of secondary metabolites, signal transduction mechanisms, and other cellular components and molecular function related terms based on their matches in the GO, COG and KEGG databases. This study demonstrated that the Illumina paired-end sequencing technology is a fast and cost-effective method for novel gene discovery in non-model plant organisms. Furthermore, radish unigenes provided a comprehensive enough coverage to allow for the discovery of almost all genes known to be involved in GS metabolism and regulation related pathways. Our transcriptome dataset will serve as a valuable public platform to enhance the understanding of molecular mechanisms underlying biosynthesis and metabolism of the nutritional and flavor components during taproot formation. It would further facilitate the genetic improvement of major quality traits in radish breeding programs.

## Methods

### Plant materials

The radish (*Raphanus sativus* L.) advanced inbred line, ‘NAU-RG’, was used in this study. The surface-sterilized seeds were sown into soil in plastic pots and the seedlings were cultured in a growth chamber with 14 h light at 25°C and 10 h dark at 18°C. For Solexa analysis and T-A cloning sequencing, taproots were sampled at three different developmental stages including seedling, taproot thickening, and mature stages. The subsamples of root, leaf and stem parts were collected at seedling, taproot thickening, and mature stages, respectively for qRT-PCR verification (the skin and flesh at mature stage were separated). All samples were washed with distilled water, immediately frozen in liquid nitrogen and stored at −80°C for RNA extraction.

### RNA extraction and Illumina sequencing

Total RNA of the three taproot samples from different stages was isolated using the RNAprep pure Plant Kit (Tiangen Biotech Co., Ltd., China) according to the manufacturer’s protocol. RNA samples were treated with RNase-free DNase I (Takara, Japan) to avoid DNA contamination. cDNA was prepared by equally pooling a total of 10 μg of RNA from each of the taproot sample of three different developmental stages. The mixed root cDNA library named ‘CKA’ was constructed using an mRNA-seq assay for paired-end transcriptome sequencing, which was performed by the Beijing Genomics Institute (BGI, Shenzhen, China).

Poly(A) mRNA was enriched from total RNA by using Sera-mag Magnetic Oligo (dT) Beads (Thermo Fisher Scientific, USA) and then mRNA-enriched RNAs were chemically fragmented to short pieces using 1× fragmentation solution (Ambion, USA) for 2.5 min at 94°C. These short fragments were taken as templates for first-strand cDNA synthesis using random hexamer-primer. The second-strand cDNA was generated using the SuperScript Double-Stranded cDNA Synthesis Kit (Invitrogen, USA). Short fragments were purified with QiaQuick PCR extraction kit and resolved with EB buffer for end repair and tailing A. Thereafter, the short fragments were connected with sequencing adapters, and the suitable fragments were selected for the PCR amplification as templates after agarose gel electrophoresis. Finally, the library was sequenced using Illumina HiSeq™ 2000.

### Raw sequence processing and *de novo* assembly

Raw reads generated by Illumina Hiseq™ 2000 were initially processed to get clean reads. Then, all the clean reads were assembled using a *de novo* assembly program Trinity [54]. Firstly, clean reads with a certain length of overlap were combined to form longer contiguous sequences (contigs), and then these reads were mapped back to the contigs. The distance and relation among these contigs was calculated based on paired-end reads, which enabled the detection of contigs from the same transcript and also the calculation of distances among these contigs. Finally, the contigs were further assembled using Trinity, and the contigs that could not be extended on either end were defined as unique transcripts. Additionally, the unigenes were divided into two classes by gene family clustering. The prefix CL was given to the clusters following the cluster id. Several unigenes with over 70% similarity were included from one cluster while from the other group the unigenes selected were singletons, for which the prefix unigene was used.

### Functional annotation and classification of the assembled transcripts

All of the assembled transcripts were compared with the publicly available protein databases including NCBI non-redundant protein (Nr), Gene Ontology (GO), Clusters of Orthologous Groups (COGs), Swiss-Prot protein and the Kyoto Encyclopedia of Genes and Genomes (KEGG), using the BLASTx analysis with a cut-off *E* value of 10^-5^. The best alignments were used to identify sequence direction and to predict the coding regions of the assembled unigenes. If the results from different databases conflicted with each other, a priority order of nr, Swiss-Prot, KEGG and COG was followed. When a unigene happened to be unaligned to none of the above databases, software ESTScan was introduced to decide its sequence direction [55]. For the nr annotations, the BLAST2GO program was used to get GO annotations of unique assembled transcripts for describing biological processes, molecular functions, and cellular components [56]. After getting GO annotations for each transcript, WEGO software [57] was used to conduct GO functional classification for understanding the distribution of gene functions at the macroscopic level.

### Gene validation by T-A cloning and sequencing

Specific PCR primers of the eight selected genes (Additional file 5) were designed corresponding to the conserved region of radish EST sequences from radish cDNA library [58]. PCR was performed in a total volume of 25 μl containing 2.0 mmol/L Mg^2+^, 0.15 mmol/L dNTPs, 0.4 mmol/L of each primer, 0.8 U Taq DNA polymerase (TAKARA) and 15 ng cDNA with the following conditions: an initial denaturation step at 94°C for 1 min, 35 cycles at 94°C for 50 s, 56°C for 50 s, and 72°C for 90 s, a final extension at 72°C for 10 min and hold at 4°C. The PCR products were separated and ligated into the pMD18-T vector (Takara Bio Inc., China), and then transformed into *E. coli* DH5α. Positive clones were sequenced with ABI 3730 (Applied Biosystems, USA).

### Quantitative real-time PCR (qRT-PCR) analysis

Quantitative real-time PCR was performed on a MyiQ Real-Time PCR Detection System (Bio-Rad) platform using the SYBR Green Master ROX (Roche, Japan) following the manufacturer’s instructions. Primers were designed using Beacon Designer 7.0 software, and Actin2/7 (*ACT*) (Additional file 6) was selected as the internal control gene [59]. Amplification was achieved by a PCR program having a first denaturation step at 95°C for 5 min, then 40 cycles of denaturation at 95°C for 5 s, followed by annealing and extension at 58°C [30, 59]. The relative expression levels of the selected transcripts were normalized to *ACT* gene and calculated using the 2^-∆∆Ct^ method. All reactions were performed in three replicates, and the data were analyzed using the Bio-Rad CFX Manager software.

### Availability of supporting data

The RNA sequence dataset supporting the results of this article is available in the [NCBI Sequence Read Archive] repository, [SRX316199 and http://www.ncbi.nlm.nih.gov/sra/].

## Electronic supplementary material

Additional file 1: **Length frequency distribution of contigs and unigenes obtained from** ***de novo*** **assembly.** (DOC 58 KB)

Additional file 2: **KEGG pathways of the assembled transcripts.** (XLS 698 KB)

Additional file 3: **Candidate genes involved in glucosinolate metabolism of radish.** (XLS 126 KB)

Additional file 4: **Candidate genes involved in MYB transcript factors.** (XLS 232 KB)

Additional file 5: **Primers used for T-A cloning and sequencing.** (DOC 38 KB)

Additional file 6: **Primers used for qRT-PCR analysis.** (DOC 32 KB)
