# Supplementary material for: De novo transcriptome sequencing of radish (Raphanus sativus L.) and analysis of major genes involved in glucosinolate metabolism
Source: BMC Genomics. 2013 Nov 27;14(1):836. doi: 10.1186/1471-2164-14-836 (PMC4046679; doi:10.1186/1471-2164-14-836)
Supplement: Supplementary file 1 — Additional file 1: Length frequency distribution of contigs and unigenes obtained from de novo assembly. (DOC 58 KB) [file 12864_2013_5529_MOESM1_ESM.doc]

A

B


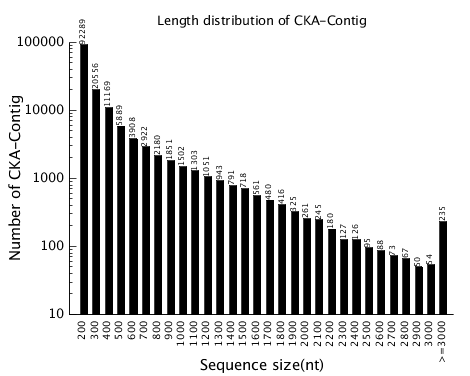


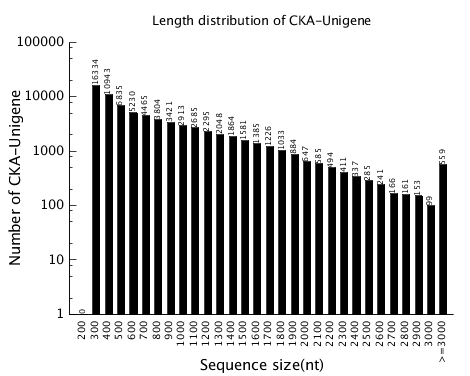


**Additional file 1** Length frequency distribution of contigs and unigenes obtained from *de novo* assembly
