# Supplementary material for: De novo transcriptome sequencing of radish (Raphanus sativus L.) and analysis of major genes involved in glucosinolate metabolism
Source: BMC Genomics. 2013 Nov 27;14(1):836. doi: 10.1186/1471-2164-14-836 (PMC4046679; doi:10.1186/1471-2164-14-836)
Supplement: Supplementary file 5 — Additional file 5: Primers used for T-A cloning and sequencing. (DOC 38 KB) [file 12864_2013_5529_MOESM5_ESM.doc]

**Additional file 5 Primers used for T-A cloning and sequencing**

| Primers | Primer sequence (5′-3′) |
| --- | --- |
| *RsBCAT4* | ACACAAAAGCAACTCGACTCAAAC |
|  | TACCAACTTTTTAAATACATCACA |
| *RsCYP79F1* | TCTCTACTCACACGCACAAACA |
|  | ACGGCGTCTTGTTTCCTT |
| *RsCYP83A1* | GGGGTAGTAACAACTAAGAA |
|  | GGAAACCAAACCAAAGAGCATAGT |
| *RsSUR1* | ACCAAAACAAAGCACAAACGAG |
|  | TGCCCCTAGACACTGGA |
| *RsUGT74B1* | TTCCTCCGTGTCTTCTTCAAC |
|  | CAACTCTTAAAACAACAACAAACA |
| *RsGS-OX1* | GAACATACAACTAAAAGAAAATAC |
|  | GAAAGTAGTAGCGATTGTATGAC |
| *RsMYB28* | GGGACCATCACACAATTCATTTCTC |
|  | TTTCTTACTTCTTGCGGTGTCTTA |
| *RsMyr1* | GACAACACAACACATACATCAAAA |
|  | CCCGAAGAGGAAAAAGTGTTTCA |
