# Supplementary material for: De novo transcriptome sequencing of radish (Raphanus sativus L.) and analysis of major genes involved in glucosinolate metabolism
Source: BMC Genomics. 2013 Nov 27;14(1):836. doi: 10.1186/1471-2164-14-836 (PMC4046679; doi:10.1186/1471-2164-14-836)
Supplement: Supplementary file 6 — Additional file 6: Primers used for qRT-PCR analysis. (DOC 32 KB) [file 12864_2013_5529_MOESM6_ESM.doc]

**Table S5 Primers used for qRT-PCR**

| Primers | Primer sequence (5′-3′) |
| --- | --- |
| *RsBCAT4* | AGTATGTATGTTGCGAAGTG |
|  | AAGGGCTGATTTGGAGAT |
| *RsUGT74B1* | TAGCGAAGTTACCAGAAG |
|  | GAGTCAAGAAACAACCTATC |
| *RsGS-OX1* | CAAGAGACATACACATAA |
|  | ATAAGAAGGAAGACTACT |
| q*RsMyr1* | TGCGAAGAGAATGAACCA |
|  | CAACACCGAAGATGAAGTC |
| *Actin2/7* | GCATCACACTTTCTACAAC |
|  | CCTGGATAGCAACATACAT |
